# Modeling the Dynamic Linkage between Tourism Development, Technological Innovation, Urbanization and Environmental Quality: Provincial Data Analysis of China

**DOI:** 10.3390/ijerph18168456

**Published:** 2021-08-10

**Authors:** Zhang Chenghu, Muhammad Arif, Khurram Shehzad, Mahmood Ahmad, Judit Oláh

**Affiliations:** 1 School of Economics and Finance, Xi’an Jiaotong University, Xi’an 710054, China; zch@mail.xjtu.edu.cn; 2 School of Economics and Management, Southeast University, Nanjing 210096, China; 233189917@seu.edu.cn; 3 Business School, Shandong University of Technology, Zibo 255000, China; mahmood@sdut.edu.cn; 4 Department of Management, Faculty of Applied Sciences, WSB University, 41300 Dabrowa Górnicza, Poland

**Keywords:** CO_2_ emission, GDP, GDP square, tourism development, technological innovation, urbanization

## Abstract

This study investigates the linkage between tourism development, technological innovation, urbanization and environmental degradation across 30 provinces of China. Based on data from 2001 to 2018, the study used an advanced economic methodology for the long-run estimate, the Augmented Mean Group (AMG) estimator, which accounts for heterogeneity in slope parameters and dependencies across countries. The empirical results show that tourism development degrades environmental quality, while technological innovation mitigates carbon emissions. Further, findings show that urbanization increases carbon emissions, while an inverted U-shaped relationship exists between economic growth and environmental degradation, implying the existence of EKC in China. Further, the Dumitrescu–Hurlin panel causality test shows that any policy aimed at tourism development or technological innovation would substantially contribute to environmental degradation, but not the other way round.

## 1. Introduction

China has acknowledged the need for all 30 provinces to minimize overall carbon emissions and energy use while prioritizing economic growth at equal intervals to provide more provisions and boost people’s quality of life. Climate change mitigation has become a significant challenge under severe environmental issues and coordinating economic growth with environmental protection [1]. The National Leading Committee on Climate Change (NLCCC) put forth the objectives and actions to reduce energy consumption and adapt to climate mitigation [2]. According to the Copenhagen summit in 2009, China has adopted a number of concrete policies, and the most important of these is that NLCCC aims to minimize intensity per unit of GDP growth by 40 to 45% in 2020, relative to 2005 levels, and to raise the proportion of non-fossil fuel in primary energy usage to about 20% by 2020 [3]. Other policies include new fossil plant construction limitations, raising provincial energy intensity standards and phasing out obsolete coal-fired power plants with a minimum capacity of less than 100 MW [4]. In addition, these policies have indeed been enacted in full compliance with the National Economic and Social Development Plans, which promote “Low-Carbon” projects for development [5]. Globally, tourism has expanded so much that it is considered to be a key economic sphere for many countries around the globe. The tourism industry has expanded vividly in China for the last few years [6]. China ranks first in the world on the contribution of travel and tourism to employment (66,086,000 employees in 2014). In 2019, China earned 6.63 trillion yuan (about $935 billion), making it among the top tourist destinations worldwide in terms of inbound travel receipts [7]. Tourism development has been somewhat asymmetrical, with regions on the east coast growing faster than others. Its contribution to GDP increased by 25% between 1995 and 2005, and according to the World Trade Organization, it has become one of the main generators of employment [8]. China’s tourism has enormous potential; as 40 of the 911 UNESCO sites worldwide are focused in the region, tourism is highly supported through national policies at the government level [9]. According to the UNWTO, tourism-related emissions allow 5% of total man-made emission in 2016 and which are expected to climb to 5.3% by 2030 under the current scenario of ambition. The predicted increase is 25%, from 1597 million tons of CO_2_ attributable to tourism-related transportation in 2016 to 1998 million tons in 2030 [10]. Due to the lack of sustainable tourism in many provinces of China, air pollution in many provinces is increasing excessively, leading to poor air quality and environmental degradation. For example, in Guangdong and Shandong, air pollution increases due to the tourism industry [11,12]. Peng and Deng [13] studied five provinces and eight cities for pilot studies on “Low-Carbon” growth under the NDRC reforms. China has achieved remarkable economic growth during the last few years, which in turn increases enormous pressure on natural resources and environmental quality. To minimize the environmental impact of economic growth, significant change in the economic structure has occurred. China is currently putting more effort into optimizing its economic structure and adjusting its industrial structure. The economic growth driver has moved from factor-driven to innovation-driven growth. The Chinese economy is characterized as somehow being heterogeneous. While the economies of the more developed eastern coastal regions have improved significantly in terms of per capita income, some of the less developed western regions continue to struggle to meet basic needs [14].

Furthermore, the disparity in income and availability of services between urban and rural areas prevail. Though, most urban areas have upgraded infrastructure and living standards. Many rural areas have poor infrastructure, such as sanitation facilities [15]. Based on these facts, the Chinese Government should take further steps to recognize the policies to take regional differences into account, impacting the actual drivers of provincial and regional CO_2_ emission. After the introduction of transparent and reform policies in 1978, China has undergone rapid economic growth. The economy is in the process of transitioning from a heavily regulated planning economy to a market economy [16].

The Chinese central government has highlighted economic cooperation among various regions while encouraging regional governments to adopt their own development policies based on their unique characteristics. Different provinces have specialized in economic activities based on their natural resource endowment, manufacturing sector, and research and development (R&D) capabilities [17]. Because of their natural resource endowment, some provinces, for example, promote the growth of heavy industry [2]. However, due to a lack of robust environmental and resource efficiency considerations, these provinces are now facing significant resource scarcity and environmental problems, such as a lack of coal tailing treatment and water or air pollution challenges. These provinces lack the human capital associated with maintaining their infrastructure and facilities, forcing them to continue relying on fossil fuels and outdated technologies to support their economies. Heilongjiang, Shanxi, Xinjiang, Inner Mongolia and Qinghai are examples of those provinces [18]. In comparison, coastal provinces typically lack substantial natural resources but have more evolved human resources, modern technology (education, transportation network, telecommunication services, and so on) and improved climate conditions. As a result, these provinces promote high-tech manufacturing and the service sector, resulting in greater sustainability.

This paper aims to provide a novel, modern approach to better understanding the role of technological innovation and tourism for environmental sustainability across provinces of China. The study also is concerned with higher urban density inclines to augmented CO_2_ emission to the level of provincial basis. This is done by investigating how these environmental-related factors respond to climate change in different regions. To accomplish this, three key objectives are drawn for investigation as mentioned below. The primary goal of this analysis is to look at the significant effect of tourism development on the level of environmental degradation; second, the effect of technological innovation upon environmental quality across the Chinese provinces; third, research the effect and relationship between urbanization and CO_2_ emission; fourth, this investigation also considers the importance of GDP and GDP square in determining the nature of the EKC curve in China’s provinces. Therefore, it is imperative to answer the following questions in the sense that tourism affects CO_2_ emissions: (i) Does technological innovation increase the quality of the environment? (ii) Does economic growth accelerate CO_2_ emissions? (iii) What effect does urbanization have upon environmental quality?

The study adds to the existing body of literature in the following ways. In this study, we investigate the impact of tourism development on environmental degradation in China. For this purpose, we measured the environmental degradation and tourism development with carbon dioxide emissions (CO_2_) and the Number of Overseas Visitor Arrivals (million person-times). Second, we include technological innovation in the model measured by the patent applications (resident + non-resident), since technological innovation can play an important role in influencing tourism development and CO_2_ emission. Moreover, urbanization is measured through resident population per 10,000 persons. Third, we employed sophisticated econometric tools, such as the Augmented Mean Group (AMG) estimator, using a panel data sample from 2001 to 2018, in contrast to earlier studies. This approach provides robust results in the presence of cross-sectional dependence, misspecification, non-stationarity, serial correlation of error term, heterogeneity and endogeneity bias problems.

The remaining paper is divided as follows: Section 2 gives a brief overview of the literature, and Section 3 includes the theoretical framework. Section 4 contains the materials and methods used in this study. Section 5 contains the discussion, and Section 6 summarizes the conclusion and presents policy recommendations.

## 2. Literature Review

Economic structure optimization and industrial adjustment are necessary for every developed nation, and China is making an effort for economic and innovation growth. According to Liu et al. [19], economic growth can be accessed by improving the total factor of production. Industrial society uses machinery for massive production that may cause serious environmental pollution. The significance of technological innovation in reducing CO_2_ emissions is critical. Many scholars have investigated the dynamic relationship between CO_2_ emissions and technological progress [20,21]. Irandoust [22] placed a high value on the dynamic link between technological innovation and CO_2_ emissions. Fan et al. [23] analyzed the technological innovation effect on CO_2_ emissions of different income countries by using the STIRPAT model from 1975 to 2000. Findings suggested that technological innovation has a different impact on different countries during the formulation of long-term policies. Zhao et al. [24] worked on Chinese power industries from 1980 to 2010 by employing the ARDL model. Results showed that technological innovation could play a critical role in reducing environmental degradation. The most essential aspect in determining environmental quality is the relationship between energy use, economic development and carbon emissions. The EKC curve is usually employed to investigate economic growth as well as environmental quality. Carbon dioxide causes climate change as well as global warming. The worldwide community is paying close attention to this issue. Zhao et al. [24] explored the causal relationships among economic growth and CO_2_ emission of 28 Chinese provinces during the year 1995 to 2007; they discovered a U-shaped connection between economic growth and CO_2_ emission. Nasir and Rehman [25] validated the presence of EKC using carbon emissions data from 1972 to 2008. Jayanthakumaran et al. [26] stated that per capita income is influenced due to CO_2_ emissions in China. Findings supported the EKC hypothesis. Many researchers have found unidirectional causality between income and emissions [27], while some studies found bidirectional causality [28].

Despite enormous amounts of literature, researchers have tried to incorporate economic development but also trade intensity and openness of a country. First, Grossman and Helpman [29] studied the impact of income and environmental degradation. No study has been done about the importance of urbanization. Due to a lack of consistency and coordination, energy technologies have increased tremendously in regional development planning. Charlier [30] explored that technology and affluence are affecting environmental factors. Kabir et al. [31] and Shehzad et al. [32] observed that carbon capture and storage technologies, renewable and clean technologies, and high-efficiency energy usage technologies are the three primary categories of low-carbon technology. Wang et al. [33] checked technology gaps and CO_2_ emission performance in China, and researchers faced difficulties in comparing the results due to period and sample differences.

Bilgili et al. [34] investigated 17 OECD countries from the year 1977 to 2010 by employing FMOLS and DOLS estimations. Their findings supported the hypothesis of EKC for the panel. Zheng et al.’s [35] high priority is clean energy supply and ignoring local consumption capacity. Chinese industries consume 70% of total energy, but the consumption in several OECD and developed countries is greater than the per household energy consumption of China. For instance, in the year 2012, Chinese household energy consumption was 4%, which is equal to 38% in the EU-27 in the year 2008. In energy infrastructure construction, western and central Chinese provinces are far behind other eastern countries. The demand for energy in the residential sector is increased dramatically due to urbanization. To overcome the increasing biological and environmental problems, researchers engaged in investigating the technological innovation impact on CO_2_ emissions. According to Kumar et al. [36], technological innovation increases CO_2_ emissions in developing nations while decreasing CO_2_ emissions in rich nations. Similarly, several researchers have looked at the link between technological innovation and CO_2_ emissions, such as Wang et al. [37] and Wang and Feng [38]. Poumanyvong and Kaneko [39] confirmed the inhibitory effect of technological innovation on CO_2_ emission in China of 99 countries from the years 1975 to 2005 by employing the STIRPAT model; results displayed the significant, negative relationship between technology and CO_2_ emission. Bilgili et al. [34] investigated Britain’s upstream industries and employed an input–output model to check CO_2_ emissions in China. These studies have supported the dynamic links between carbon emissions and advancement in technology. CO_2_ emissions are also rising due to traditional technology, urbanization, the possible number of overseas arrivals and, most significantly, unsustainable, rapid economic development patterns.

On the other hand, the link between technological advances, tourism development, urbanization and CO_2_ emissions across China’s provinces is not adequately illustrated in the current literature. Therefore, the study considered that technological innovation on CO_2_ emissions in China’s environment has not been actively investigated. Wang et al. [40] stated that urban areas have educational resources, such as research institutions and universities, that can conduct R&D and cultivate personnel. This is why urbanization promotes energy savings and technologies. According to Song et al. [41], urbanization is not increasing at the same rate, while CO_2_ emissions have been growing rapidly since 2000 in the YRD. The urbanization level has increased from 47.95% to 65.09% over ten years. The urbanization level of Zhejiang and Jiangsu is less than that of Shanghai province. The Jiangsu urbanization level is the lowest but increases day by day. The urbanization gap between Zhejiang and Jiangsu has narrowed.

Additionally, Hao et al. [42] reported middle-income countries like China where urbanization, economic growth, and industrialization have contributed to augmented CO_2_ emissions. There is a U-shaped curve between urbanization and CO_2_ emissions; at the early stage of urbanization, efficiency of CO_2_ emission declines, and it increases when urbanization extends to a longer span. Urbanization has a negative impact on CO_2_ emissions. Poumanyvong and Kaneko [39] classified the impact of urbanization into three concepts: compact city, urban environmental transformation, and ecological modernization. Madlener and Sunak [43] outlined the impact of urbanization on energy consumption, which affects carbon dioxide emissions. Furthermore, the study found that the impacts varied between industrialized and developing nations. Wang et al. [44] investigated the Granger causality framework. The Granger causality effect was frequently used to examine the link between emissions and urbanization, and it provided that urbanization does not cause carbon emissions in BRICS countries. Extensive research has focused on developing countries. The study of Shahbaz et al. [45] proposed an inverted U-shaped relationship between carbon dioxide emissions and Malaysian urbanization. The study found a rising influence of urbanization on CO_2_ emissions using Pakistan’s time-series data. According to Sharma [46], uncovered low-, middle- and high-income nations are negatively associated with urbanization and carbon dioxide emissions.

Tourism development has a multifaceted influence on CO_2_ emissions and economic growth via numerous networks. Katircioglu [47] explored the long-run magnitude and causal linkages between tourism and GDP growth for many countries. The investigation also found another relationship, where tourism and economic growth have a one-way causal interaction in Cyprus. Khan et al. [48] supported the unidirectional causality of tourism, while bidirectional causality can be measured through the Croux–Roesens causality test. Results showed that tourism is the key determinant to increase CO_2_ emissions and economic growth. After reviewing the literature, we can conclude that research on the link between tourism development and CO_2_ emissions is accessible; however, the results are contradictory. Furthermore, none of the studies examined the role of technological innovation, which might play a key role in attaining sustainable development. In this context, the current study investigates the EKC hypothesis’s link between tourism development, technological innovation, economic growth and CO_2_ emissions.

## 3. Theoretical Framework

This section covers the theoretical framework through the impact of economic growth, tourism development and technological innovation on environmental quality. In the prevailing literature, the Environment Kuznets Curve (EKC) approach is extensively used to examine the environmental impact of economic growth. In 1991, Grossman and Krueger [49] first used the concept of the EKC to study the impact of economic growth on environmental quality. According to the EKC hypothesis, environmental pressures increase as income level increases at the initial stage of economic development, but later these pressures decrease when income level reaches a certain level. Economic growth can affect the environmental quality through three main channels: scale, composition, and technique effects [50]. The scale effect is associated with higher demand without a change in technology or economic conditions. Thus, economic development seems to degrade environmental quality, as more outstanding manufacturing requires more raw materials to boost economic activities, which produces more waste and emissions [51]. Moreover, the composition impact exposes the rate of pollution as well as the materials utilized in manufacturing depending on an economy’s sectoral structure. It is natural for emerging economies to experience systemic shifts. Transitions from the agriculture to the manufacturing sectors, and subsequently from the manufacturing to the service sectors, may be included. In this scenario, the formulation reduces the quantity of resources used and helps reduce the negative environmental impacts that arise from rapid economic expansion. If the structural change is sufficiently big in scope, the compositional influence would also reduce the more scalable economic growth impact [52,53]. Furthermore, the third channel to be considered is the technical impact. The technical impact helps to increase efficiency and to implement new technology that is sophisticated and clean. These new technologies will eventually assist to limit the deterioration of the environment, which poses a serious threat around the world [20,21,54].

In addition to economic growth, tourism can also affect environmental quality. The relationship between tourism and environmental quality is complex, and many activities adversely impact ecological quality. For instance, the development of the tourism sector is linked with the construction of infrastructure (tourism facilities, hotels, roads and airports), which negatively affects the environmental quality [55]. On the contrary, tourism can be beneficial for environmental protection and conservation. Tourism may create awareness of environmental values, and it can serve as a tool to finance the protection of natural areas [56]. Theoretically, a systems approach is beneficial for understanding the dynamics of tourism and the environmental quality. Moreover, it also highlights the interdependence of ecosystem species and how, like a spider web, touching one component causes a ripple effect across the whole [57]. The idea of a systems approach is to investigate nature holistically, i.e., the connection of the components, instead of using a reductionist approach that focuses on one specific element [58]. The carbon cycle is an example of systems thinking applied to nature, and its unbalance is a contributing cause to rising levels of CO_2_ in the atmosphere, which is a key factor in global climate change. Comprehensive thinking and a collaborative approach are required in order to construct a sustainable tourism sector [59]. Endogenous growth theory and ecological modernization theory support the idea that, with the help of technological innovation, countries can achieve sustainable economic growth without hurting the environmental quality.

## 4. Material and Methods

### 4.1. Data and Variables

The current study focused on 30 Chinese provinces (excluding Tibet, Hong Kong, Macao and Taiwan). The usage of fossil fuels significantly increases CO_2_ emissions. The fuel energy use statistics for 30 Chinese provinces utilized in this research were obtained from the China Energy Statistical Yearbook. The panel data from 2001 to 2018 were used in this study analysis on an annual basis. The remaining variables were taken from the World Development Indicator, which include GDP, GDP squared, tourism development, technical innovation and urbanization. Data sources and units are provided in Table 1.

Based on the theoretical explanation, this study developed the following model.

(1) $$Y=f\;\left(\mathrm{GDP},{\;\mathrm{GDP}}^{2},\;\mathrm{TD},\;\mathrm{TI},\;\mathrm{UR}\right)$$

*Y* represents carbon dioxide emissions; GDP per capita and GDP squared are included; TD represents tourist development; TI represents technical innovation; UR represents urbanization in this equation. Provinces in China are divided into three areas to examine the geographic origins of provincial CO_2_ emission levels: eastern, central, and western. Beijing and Tianjin are the official regional classifications of the Chinese government. The eastern region includes Beijing, Tianjin, Hebei, Shanghai, Jiangsu, Zhejiang, Fujian, Shandong, Hainan and Liaoning. Jilin, Heilongjiang, Shanxi, Anhui, Jiangxi, Henan and Hunan are part of the central region. Inner Mongolia, Guangxi, Chongqing, Sichuan, Guizhou, Yunan, Shaanxi, Hubei and Hunan are part of the western region.

### 4.2. Econometric Methodology

While developing the econometric methodology, this analysis takes into account sophisticated econometric approaches. This methodology is split into six steps: (a) first, a study of common stock effects using the cross-sectional dependence test [60]; (b) second, a slope of homogeneity test in panel data using the heterogeneity test [61]. (c) Third, the CIPS unit root test of [52] is used to examine the stationary properties of the individual variables; (d) fourth, the [62] panel cointegration test is used to assess the equilibrium connection among the variables. (e) Fifth, then the Eberhardt [63] test of Augmented Mean Group (AMG) is utilized to examine the robustness.

#### 4.2.1. Cross-Sectional Dependence Test

Initially, in this research, the cross-sectional dependency (*CD*) test was utilized to assess whether or not *CD*s occur in the dataset in terms of the estimation of the variables used in this study. The *CD* assessment is conducted systematically, beginning with the panel data analysis. Acknowledging the *CD* in panel data is important since the CIPS and Westerlund cointegration methods use second-generation unit tests and presume dependence and heterogeneity among separate parts. In order to get the most trustworthy and viable data, the current study applies the Pesaran [60] cross-section dependence (*CD*) test.

During the computation of the correlation variables, it eliminates the values of the means. In contrast to the alternative hypothesis, which asserts that the *CD* is present in the proof, the null hypothesis asserts that the *CD* is not there. The *CD* assessment is as follows:

(2) $$CD=\sqrt{\frac{2T}{N\left(N-1\right)}}\left(\overset{N-1}{\underset{i=1}{\sum }}\overset{N}{\underset{j=i+1}{\sum }}{\hat{\rho }}_{ij}\right)$$

#### 4.2.2. Slope of Homogeneity Test

In the study, the slope homogeneity test devised by Pesaran and Yamagata [61] was utilized to demonstrate slope heterogeneity between the cross-sectional units. In addition, the slope homogeneity test is preferred to other common homogeneity tests. Further to that, the slope homogeneity test outperforms other common homogeneity tests. As an example, these seem to be unrelated regression equations.

(3) $${\tilde{\Delta }}_{SH}={\left(N\right)}^{\frac{1}{2}}{\left(2K\right)}^{-\frac{1}{2}}\left(\frac{1}{N}\tilde{S}-k\right)$$

(4) $${\tilde{\Delta }}_{ASH}={\left(N\right)}^{\frac{1}{2}}{\left(\frac{2k(T-k-1}{T+1}\right)}^{-\frac{1}{2}}\left(\frac{1}{N}\tilde{S}-k\right)$$

#### 4.2.3. Unit Root Test

After reviewing the *CD* and slope homogeneity measurements, the following step is to evaluate the path of cointegration of the variables under consideration in this research. The consequences of the first-generation unit root test might be biased if the correctional units are independent [54]. To prevent bias in their results, Danish et al. [51] suggested using parametric and non-parametric assessments. The consequence is that, in this analysis, the unit root test and cross-sectionally Augmented IPS (CIPS) tests were used [64]. Furthermore, the CIPS could be more responsive to *CD* and variability, thereby being more suitable for the research. Consequently, the CIPS test equation is as follows:

(5) $$\Delta CAS_{i,t}=\varphi_{i}+\varphi_{i}Z_{i,t-1}+\varphi_{i}{\bar{CAS}}_{t-1}+\overset{p}{\underset{l=0}{\sum }}\varphi_{il}\Delta \bar{CAS_{t-1}}+\overset{p}{\underset{l=0}{\sum }}\varphi_{il}\Delta CAS_{i,t-1}+\mu_{it}$$

where $\Delta \bar{CAS_{t-1}}$ and ${\bar{CAS}}_{t-1}$ are the average cross-sections. The research elucidates the CIPS test statistics as follows:

(6) $$\hat{CIPS}=\frac{1}{N}\overset{n}{\underset{i=1}{\sum }}CDF_{i}$$

The cross-sectional Augmented Dickey–Fuller is denoted by *CDF* (CADF).

#### 4.2.4. Westerlund Test for Panel Cointegration

Prior to estimating the long-run parameters, the study confirms whether cointegration exists among the underlying variables. The panel cointegration Westerlund test is included in this analysis [62]. It offers consistent and trustworthy data and supports monitoring the error term’s cross-sectional dependency [65]. The null hypothesis cointegration test will presume that no cointegration exists for at least one cross-section of Gt and all cross-sections of Pt. Subsequently, the Westerlund cointegration equation is written as follows:

(7) $$\Delta Y_{t}=\delta_{i}^{\prime }d_{t}+\alpha_{i}Y_{i,t-1}+\lambda_{i}^{\prime }X_{i,t-1}+\overset{p_{i}}{\underset{j=1}{\sum }}\alpha_{ij}\Delta Y_{i,t-1}+\overset{p_{i}}{\underset{j=-qt}{\sum }}\gamma_{ij}\Delta X_{i,t-1}+u_{it}$$

where $\delta_{1i}=\alpha_{i}\left(1\right)\phi_{2i}-\alpha_{i}\phi_{1i}+\alpha_{i}\phi_{2i}$ and $\delta_{2i}=-\alpha_{i}\phi_{2i}$.

In Equation (7), β is an error correction coefficient, and $\alpha_{i}$ is the vector of the x and y cointegration relationship. The test statistics are below:

(7a) $$G_{t}=\frac{1}{N}\overset{N}{\underset{i=1}{\sum }}\frac{\alpha_{i}^{\prime }}{SE\left(\alpha_{i}^{\prime }\right)}$$

(7b) $$G_{a}=\frac{1}{N}\overset{N}{\underset{i=1}{\sum }}\frac{T\alpha_{i}^{\prime }}{\alpha_{i}^{\prime }\left(1\right)}$$

(7c) $$P_{t}=\frac{\alpha^{\prime }}{SE\left(\alpha^{\prime }\right)}$$

(7d) $$P_{a}=T\alpha^{\prime }$$

(7e) $$\alpha^{\prime }=\frac{P_{a}}{T}$$

where $G_{t}$ and $G_{a}$ indicate the group means’ statistics. $P_{a}$ and $P_{t}$ represent the panel statistics. The parameter for the error correction $\left(a^{\prime }\right)$ in Equation (7) can be calculated by putting the value of $P_{a}=Ta^{\prime }$ in Equation (7d). Therefore, the error correction parameter is $a^{\prime }=\frac{P_{a}}{T}$, which is the amount of error to be corrected annually, in case short-term disparity occurs.

#### 4.2.5. Augmented Mean Group Estimation

The Augmented Mean Group (AMG) methodology pioneered by Eberhardt [63] is utilized in this research, which tackles the issues of non-stationarity, endogeneity and slope heterogeneity problems and provides reliable results. The AMG estimation equation is shown.

(8) $$\Delta CO_{2}{}_{it}=\beta_{1}\Delta GDP_{it}+\beta_{2}\Delta GDP_{it}^{2}+\beta_{3}\Delta TD_{it}+\beta_{4}\Delta TI_{it}+\beta_{5}\Delta URB_{it}+\overset{T}{\underset{t=2}{\sum }}c_{t}\Delta D_{t}+\varepsilon_{it}$$

where $\Delta D_{t}$ signifies the first difference, *j* is the dummy time parameters, and *T*−1 is the period dummies. When P_t_ is replaced with *r*, the typical dynamic mechanism is revealed as follows:

(9) $$y_{it}=\theta_{i}+\beta^{\prime }x_{it}+c_{i}t+d_{i}{\hat{\phi }}_{t}+\varepsilon_{it}\Delta {\hat{\beta }}_{AMG}=N^{-1}\underset{i}{\sum }{\hat{\beta }}_{i}$$

#### 4.2.6. Panel Granger Causality Test

While the panel AMG and CCEMG methods have long-run parameters, these do not provide a causality direction between variables. Consequently, the Granger causality test developed by Dumitrescu and Hurlin [66] was used in this study to examine causal relations and the directional flow of variables. In contrast to the alternate hypothesis, which is the existence of a causal interaction between variables, the null hypothesis of D-H causality claims that no causal relationship is observed between variables. This is how the model can be expressed:

(10) $$z_{i,t}=\alpha_{i}+\overset{p}{\underset{j=1}{\sum }}\beta_{i}^{j}z_{i,t-j}+\overset{p}{\underset{j=1}{\sum }}\gamma_{i}^{j}T_{i,t-j}$$

$\beta_{i}^{j}$ represents the autoregressive parameters, and $\left(j\right)$ indicates the lag length.

#### 4.2.7. Spatial Distribution Analysis

**Figure 1:**
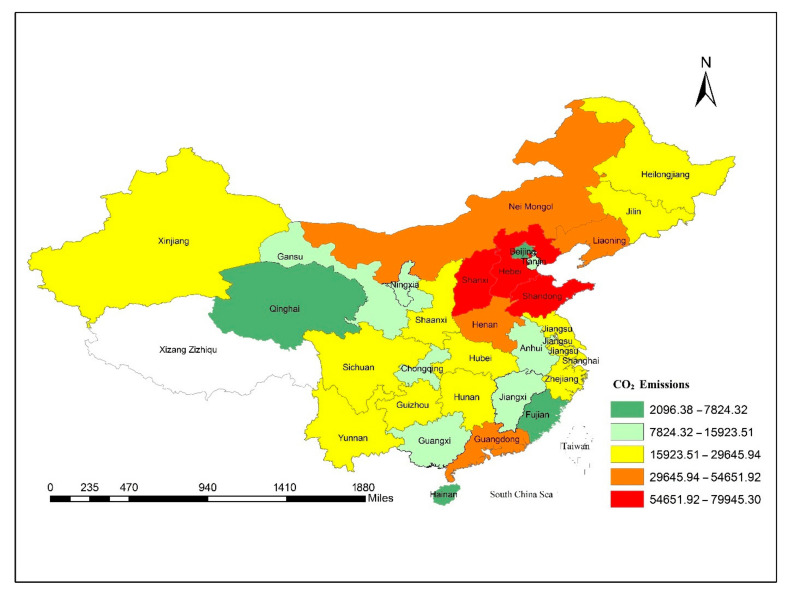
Spatial distribution of carbon dioxide emissions across the provinces of China, 2000–2018.

**Figure 2:**
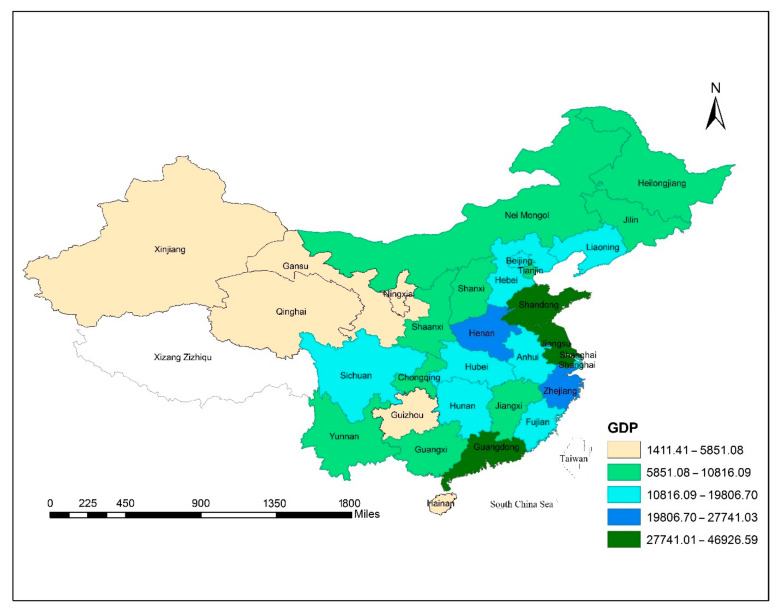
The spatial distribution of GDP per capita in China provinces from 2001 to 2018.

**Figure 3:**
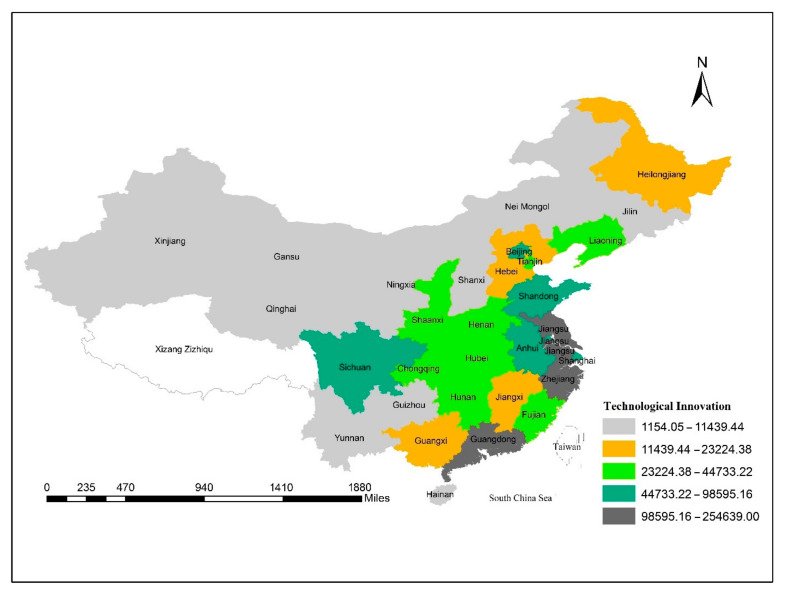
Spatial distribution in technological innovation across provinces of China, 2001–2018.

**Figure 4:**
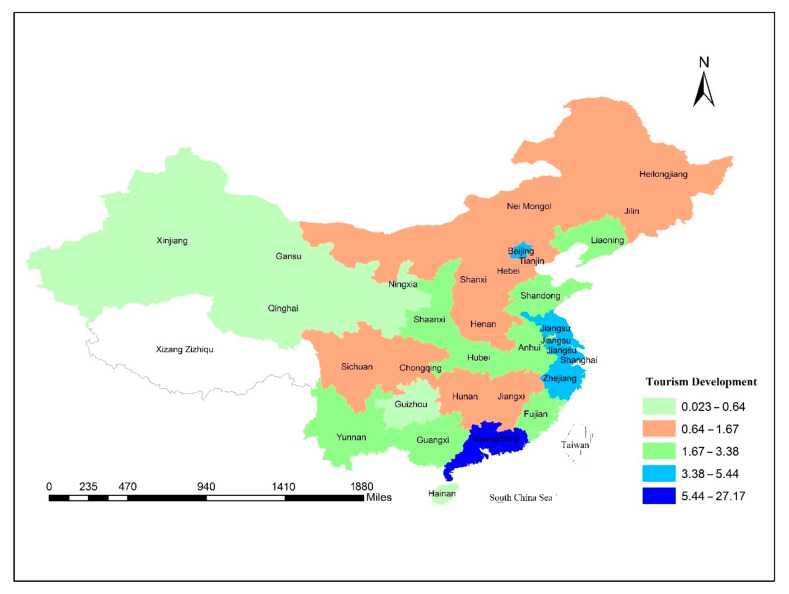
Spatial distribution of the percentage of overseas arrivals across provinces of China, 2001–2018.

Figure 1, Figure 2, Figure 3 and Figure 4 depict the spatial distribution of CO_2_ emission (in metric tons), GDP per capita, technological innovation and tourism development (percent of number of overseas arrivals in China). Figure 1 demonstrates the spatial distribution of CO_2_ emissions across the provinces of China, which clearly shows the most polluted area across China’s provinces. As can be seen in the map, Shanxi province emits higher CO_2_ emissions into the atmosphere. Henan emits less emissions. Similarly, Hebei, the third province in the North Coastal, and Shandong, the fourth province, have large emissions across China’s provinces.

Figure 2 illustrates the geographical distribution of GDP per capita in China’s provinces. In terms of GDP per capita, Shanghai, Jiangsu and Guangdong are at their peaks. Shanghai’s GDP per capita exceeded US $10,000 in 2020, growing from US $1000 in 2001. Likewise, Guangdong recorded US $5000, and Tianjin exceeded up to US $13,000. Henan and Zhejiang exceeded US $8000 and US $15,000, respectively. Guangdong and Jiangsu were estimated to generate over 10 trillion yuan ($1.45 trillion) with their respective GDP for the first time in China, where most provinces have downgraded their economic growth targets to China news service. So, Guangdong and Jiangsu took the land. Other provinces still made breakthroughs in GDP growth.

Figure 3 exhibits the spatial distribution of technological innovation across the provinces of China. As can be seen, although the Eastern Coastal area is advanced in indigenous innovation and R&D, the North Coastal and Southwest areas are on their way to catching up.

Figure 4 shows the geographical spread of overseas arrivals through China’s provinces, with Guangdong being one of the country’s most popular tourist destinations. It mostly encircles parts of the Pearl River Delta and Southeast Asia. Guangdong tourism has been expanded to remote domestic cities such as eastern China, Zhejiang, Jiangsu and Shanghai after vigorous growth. Several European and American business travelers are fascinated by these provinces. In successive years, the province’s tourism revenue has seen rapid growth. Tourism is becoming increasingly evident in the industry’s urban economic growth.

## 5. Empirical Statistical Results and Discussion

This section summarizes the study’s findings. Table 2 shows the comparative descriptions of the variables used in each of China’s 30 provinces from 2001 to 2018. The results showed that economic growth (quadratic term of GDP) had the highest mean value of 15.826, and it increased from 6.137 to 24.88 over the years from 2001 to 2018. The value of TI increased from 2.093 to 5.9, with a mean value of 4.142. The value of CO_2_ emissions increased from 2.29 to 5.11, with an average value of 4.3.

Table 3 shows that GDP and GDP squared had a strong and positive association with CO_2_ emissions. When compared to other factors that had a positive association with overall CO_2_ emissions, such as TI and TD, URB had a poor and positive correlation with CO_2_ emissions in China.

The cross-dependence (*CD*) analysis is the initial stage in the panel results analysis, and we applied the *CD* test utilized for this purpose [51]. The prevalence of cross-sectional dependence in the panel results can be seen in Table 4. Furthermore, the correlation coefficient ranges from 0.931 to 0.994, demonstrating the presence of a cross-sectional unit interaction.

Table 5 shows that the model’s coefficients are heterogeneous, and the slope changes across cross-sections due to the existence of heterogeneity, which further demonstrates that the dynamic socioeconomic relations of one region cannot be affected directly by the hierarchical socioeconomic relations of other regions [58]. The presence *CD* and a heterogenous slope in panel data require an appropriate unit root that provides robust results. Therefore, this study employed the CIPS unit root test for this specific reason. Particularly, in comparison to the traditional unit root test, the CIPS test findings are more imperative since *CD* and heterogeneity factors have more accurate results.

Table 6 represents the CIPS unit root test results, indicating that variables had a stationary problem at the level 1(0). However, at the first difference, all variables became stationary, enabling us to use cointegration techniques and the AMG methodology.

Table 7 displays the results of the Westerlund panel cointegration test. As cointegration results, the panel statistics (*P_t_, P_a_*) and group statistics (*G_t_, G_a_*) are determined. The null hypothesis was necessary to dismiss due to the cointegration that exists. In other words, the test results showed a cointegration relationship between CO_2_ emissions, economic growth, tourism development, technological innovation and urbanization. The clarification of cointegration indicates that the variables of concern tended to have a long-term relationship. In addition, in Equation (10), the error correction parameter (a‘) was $\alpha^{\prime }=\frac{P_{a}}{T}$ = −11.06/18 = −0.616 in the model. This value suggests that there is a correlation of around >61% error between CO_2_ emissions and their determinants every year.

Table 8 shows the findings of AMG empirical investigations, which show that GDP had a strong and positive influence on CO_2_ emissions; however, the GDP quadratic term had a negative influence on the environment. The presence of an Environmental Kuznets Curve in China is demonstrated by the positive sign of GDP and the negative sign of GDP squared. Notably, a 1% increase in GDP caused a 1.66 exponential increase, even if a 1% increase in GDP squared causes a −0.074 variation in CO_2_ emissions. Technological innovation can boost economic growth and tourism development in China, thereby promoting long-term development. However, because of a shortage of R&D expenditures in China, the optimal degree of technological innovation has not been achieved. Accordingly, certain advancements in technology soon will have a substantial impact on environmental efficiency. The use of TI had a negative effect on CO_2_ emissions. For AMG and CCEMG, a 1% boost in TI significantly reduced carbon emissions by −0.043. A favorable mechanism of technological innovation in decreasing CO_2_ emissions at the provincial level in China is related with technological research and education that enhances environmental and pro-environmental initiatives. TD positively affected CO_2_ emissions, where a one percent increase in TD led to a 0.038 increase in CO_2_ emissions. Similarly, urbanization also had a positive impact on CO_2_ emissions, which increased 0.404 variations in CO_2_ emissions with one percent variations in URB.

**Figure 5:**
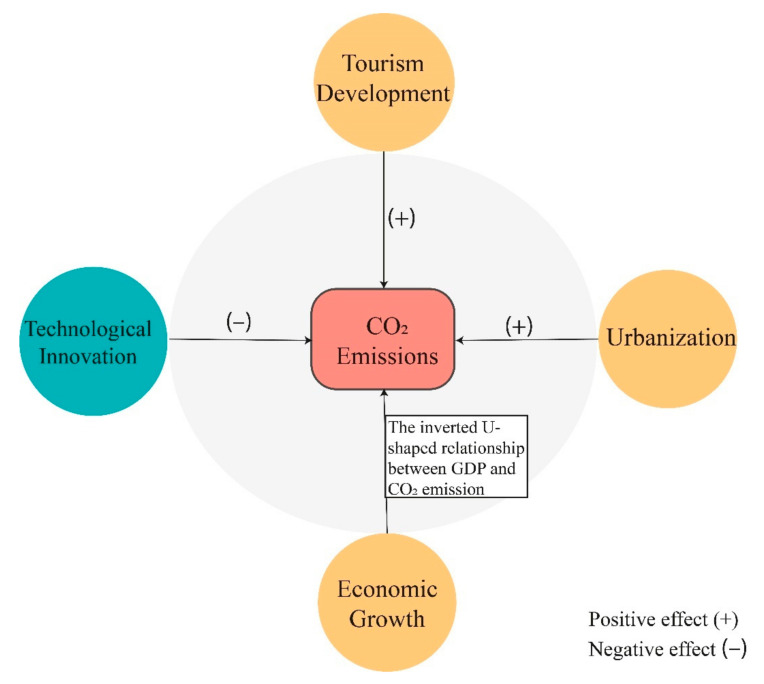
Graphical representation of results based on AMG and CCEMG estimator.

The robustness of the model was evaluated using Pesaran [67] Common Correlated Effect Mean Group (CCEMG) estimate. Table 8 shows the CCEMG results and where the results acquired by the CCEMG approach are consistent with the results obtained by the AMG approach. Economic growth, square of economic growth, tourism development, technological innovation and urbanization had a long-run elasticity of 1.837%, −0.152%, 0.059%, −0.135% and 0.233%, respectively. Figure 5 depicts the AMG and CCEMG method results graphically.

In this analysis, the CCEMG approach is used to assess the model’s robustness. As shown in Table 9, GDP had a positive coefficient, and GDP squared had a negative coefficient, which confirms the existence of the EKC hypothesis. Tourism growth had a significant and positive coefficient, whereas technological innovation’s effect on CO_2_ emissions had a statistically significant, negative value. Urbanization had a statistically significant and positive impact on CO_2_ emissions. According to the AMG estimation, the long-term elasticity of GDP, quadratic term of GDP, tourism development, technological innovation, and urbanization were 1.83, −0.152, 0.059, −0.135 and 0.233, respectively.

The findings of the Dumitrescu–Hurlin granger causality test, given in Table 10, show that economic growth, tourism development and technological innovation had causal effects on CO_2_ emissions in China. This implies that a policy to target tourism development and technological innovation will affect the environmental quality. Conversely, any policy aimed at enhancing environmental quality does not affect these indicators. Further, in the case of economic growth, urbanization and environmental pollution, bidirectional causality persists. It means that any policies aimed at promoting economic development and urbanization would have an effect on environmental quality and vice-versa.

## 6. Discussion

The findings concur with earlier obtained results highlighting the statistically positive effect of economic growth on CO_2_ emission. The deteriorating impact of GDP on environmental quality is plausible regarding China’s rapid rise; GDP has risen dramatically in recent decades as well. The GDP quadratic term is statistically negative, confirming the prevalence of the EKC hypothesis in China’s provinces. To put it another way, the overall provincial emissions are a result of China’s economic growth. Our findings are consistent with the results of Yang et al. [68]. The coefficient TD is strongly positive, indicating that a vastly increased a number of foreign visitors or tourists in China is continuing to increase total carbon emissions. The study’s findings are consistent with Jebli et al. [69], who discovered that tourism development degrades the environment and tends to increase CO_2_ emissions in Tunisia. The fact that the coefficient of technological innovation is negative indicates the strong association between technological innovation and overall carbon emissions in China. It clearly indicates that reducing technological innovation would impact total carbon emissions. This outcome is comparable to Poumanyvong and Kaneko [39], and the coefficient of urbanization is strongly positive, confirming that high urban density increases carbon emissions throughout Chinese regions. However, urbanization leads to fast economic development in infrastructure investment and people movement, which contributes to pollution [20,70]. These findings are in line with [45]. These findings imply that, initially, economic growth promotes and accelerates carbon emissions and that, after a given period, the curve begins to show a downward tendency. These empirical data validate the findings of Rehman and Rashid [71] and Rahman [72]; these all support the EKC theory, which links economic growth to CO_2_ emissions. However, several investigations, such as Ozcan [73] and Rahman [74], found contradictory results. The impact of the error correction term mostly had a positive association with tourism development and CO_2_ emissions values. This also entails that policies concerning environmental degradation, economic growth, technological innovation, tourism development and urbanization would be entirely absorbed and permeated into the environment after a year and a month or two of lag time. The AMG approach is used in this analysis to measure the model’s robustness. Table 7 shows a positive GDP coefficient and negative GDP squared. Furthermore, it lends support to the EKC hypothesis. Tourism development has been demonstrated to have a substantial and positive influence.

## 7. Conclusions and Policy Recommendations

This investigation utilizes a second-generation panel cointegration methodology to provide a dynamic linkage on the impact of tourism, innovation and urbanization on carbon emission across China’s 30 provinces from the period 2001 to 2018. Further, the research employed a spatial distribution analysis to distinguish individual provinces from other provinces in China. Further to that, the CIPS unit root test outcome demonstrates that almost all of the variables under consideration are stationary at the first difference, enabling the use of cointegration and Augmented Mean Group. The empirical data prove that technological innovation has a wide impact on carbon emissions across all of China’s provinces. Furthermore, the positive coefficient of urbanization suggests that increasing urban density boosts carbon emissions across Chinese regions. Nonetheless, urbanization causes significant economic growth in infrastructure expenditures and population movement, both of which add to emissions. According to the research, the relationship between economic growth and urbanization is quite significant. Meanwhile, urbanization has been acknowledged as a major long-term and healthy growth driver. Economic growth, from the other way, leads to population agglomeration and the synthesis of economic activities on a vast scale. In addition, the link between carbon emissions and economic growth is compatible with Environment Kuznets theory. The association between urbanization and environmental emissions was, likewise, an inverted U-shaped in the opposite direction. The research also discovered that technological innovation is strong and does play a significant role in innovation-driven economic growth. While TI and TD have a positive relationship with overall CO_2_ emissions, URB has a negative relationship with CO_2_ emissions in China. The negative coefficient of technological innovation implies that less technological innovation tends to reduce carbon emissions. The positive tourism development coefficient validates that strong tourism development is connected with emissions and concluded that a rise in tourist activities corresponds with variations in climate through multiple means, such as a growth in tourism activities and boosting energy demand in diverse functions, which is also primarily attributable to environmental worsening and carbon pollution.

### Policy Recommendation

Based upon our results and conclusions, we recommend the following policy suggestions to make China’s government help mitigate climate change even further.

Fuel and high-emission sectors, such as the manufacturing and iron and steel metal industries, should be narrowed down. Moreover, strict rules and regulations should be maintained for industrial waste. Policies such as electricity charges and environmental charges are examples of feasible policies. Meanwhile, actions must be done to assist competitive and new-technology sectors (such as digital electronics and agricultural biological industries), and China provinces should enable and promote the growth of their tertiary sectors based on their strengths (such as advanced modern service industries). Shanghai has the edge of offering a greater skillset and drawing international investment, which also creates circumstances for the growth of high-tech industrial sectors; in Hainan Province, a good aquatic environment provides the foundation for the rise of tourism and sophisticated service industries. The most important issue of concern mentioned above is energy consumption, which the government should significantly reduce. Implementing new foreign technology to significantly improve investment in energy exploitation and transition is relevant and is a sustainable way of further improving energy use performance; such initiatives (such as taxation and subsidies) should be put in place to fund those initiatives. The provincial and national governments of China, particularly in the eastern provinces, must take action to promote low-carbon tourism in an industrial atmosphere.

## Figures and Tables

**Table 1:** Variable’s measurement, symbol, and data source.

Variables	Symbol	Measurement	Data Source
CO_2_ emissions	CO_2_	Total annual emissions (metric tons)	CESY
Economic growth	GDP	GDP per capita (Constant 2003 prices in RMB)	CPSY
Tourism development	TD	Number of Overseas Visitor Arrivals (million person-times)	CPSY
Technological innovation	TI	Patent applications	CPSY
Urbanization	URB	Urban Population refers to all people residing in cities and towns (10.000 persons)	CPSY

Note: CESY—China Energy Statistical Yearbook (https://data.cnki.net/Yearbook/ (accessed on 20 June 2021)), CPSY—Chinese Provincial Statistical Yearbook (http://www.stats.gov.cn/tjsj/ndsj/ (accessed on 20 June 2021)).

**Table 2:** Descriptive statistics.

Variable	Obs	Mean	Std Dev	Minima	Maxima
*log*CO_2_	540	4.270	0.411	2.286	5.112
*log*GDP	540	3.949	0.480	2.477	4.988
*log*GDP^2^	540	15.826	3.705	6.137	24.88
*log*TD	540	0.035	0.648	−2.0	1.628
*log*TI	540	4.142	0.741	2.093	5.900
*log*URB	540	1.692	0.124	1.357	1.852

Note: Obs is the number of observations used, Std is the standard deviation, and minima and maxima are the minima and maxima values.

**Table 3:** Pairwise correlations.

Variables	CO_2_	GDP	GDP^2^	TD	TI	URB
*log*CO_2_	1.000					
*log*GDP	0.715 *	1.000				
*log*GDP^2^	0.699 *	0.997 *	1.000			
*log*TD	0.430 *	0.787 *	0.778 *	1.000		
*log*TI	0.569 *	0.943 *	0.945 *	0.763 *	1.000	
*log*URB	0.158 *	0.507 *	0.512 *	0.476 *	0.613 *	1.000

* shows significance at the level of 0.1.

**Table 4:** Correlation dependence test.

Variable	*CD*-Test	*p* Values	Abs (Corr)
*log*CO_2_	75.674 ***	0.000	0.931
*log*GDP	87.943 ***	0.000	0.994
*log*GDP^2^	87.881 ***	0.000	0.993
*log*TD	63.666 ***	0.000	0.795
*log*TI	85.641 ***	0.000	0.968
*log*URB	86.883 ***	0.000	0.980

Note: *** *p* < 0.01.

**Table 5:** Slope of homogeneity test results.

Test	Test-Stat	*p* Values
$\tilde{\Delta }$	10.615 ***	0.000
${\tilde{\Delta }}_{adjusted}$	11.539 ***	0.000

Note: *** *p* < 0.01.

**Table 6:** Results of the unit root test panel.

Variables	Level		1st Difference		
	Intercept	Intercept and Trend	Intercept	Intercept and Trend	Order
*log*CO_2_	−1.649	−2.082	−4.298 ***	−4.393 ***	I(1)
*log*GDP	−1.277	−1.943	−2.748 ***	−3.314 ***	I(1)
*log*GDP^2^	−1.155	−1.866	−2.667 ***	−3.282 ***	I(1)
*log*TD	−1.734	−2.356	−3.540 ***	−3.461 ***	I(1)
*log*TI	−1.454	−1.784	−3.226 ***	−3.751 ***	I(1)
*log*URB	−1.484	−1.833	−3.428 ***	−3.920 ***	I(1)

Note: *** *p* < 0.01.

**Table 7:** Westerlund cointegration test fallouts.

Test	Value	*Z*-Value	*p* Value
*G_t_*	−2.544 ***	−3.030	0.001
*G_a_*	−10.509	−1.209	0.113
*P_t_*	−13.383 ***	−3.873	0.000
*P_a_*	−11.066 ***	−5.095	0.000

Note: *** *p* < 0.01.

**Table 8:** Augmented Mean Group (AMG) test results.

Variables	Coefficient	Std. Error	Z-Statistics	*p* Values
*log*GDP	1.667 ***	0.480	3.48	0.001
*log*GDP^2^	−0.074 ***	0.028	−2.66	0.008
*log*TD	0.038 **	0.017	2.22	0.026
*log*TI	−0.043 **	0.021	−2.11	0.035
*log*URB	0.404 ***	0.145	2.79	0.005
C	−0.698	2.594	−0.27	0.788
Wald	36.35 ***			
RMSR (sigma)	0.0732			

Note. RMSR stands for root-mean-squared error, ** *p* < 0.05, *** *p* < 0.01.

**Table 9:** Robustness test (CCEMG).

Variables	Coefficient	Std. Error	Z-Statistics	*p* Values
*log*GDP	1.837 ***	0.447	4.11	0.000
*log*GDP^2^	−0.152 **	0.055	−2.76	0.010
*log*TD	0.059 **	0.027	2.22	0.027
*log*TI	−0.135 **	0.048	−2.80	0.035
*log*URB	0.233 ***	0.060	3.86	0.000
Constant	−0.672 *	0.400	−1.68	0.093

Notes: * *p* < 0.1, ** *p* < 0.05, *** *p* < 0.01.

**Table 10:** Dumitrescu–Hurlin panel causality results.

Null Hypothesis	W-Stat.	Zbar-Stat.	Prob.	Conclusion
*logCO_2_* does not homogeneously cause *logCO_2_*	6.276 ***	14.876	0.000	*log*GDP 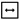 *log*CO_2_
*logCO_2_* does not homogeneously cause *logGDP*	2.533 ***	3.891	0.000	
*logTD* does not homogeneously cause *logCO_2_*	3.611 ***	7.119	0.000	*log*TD→*log*CO_2_
*logCO_2_* does not homogeneous cause *logTD*	1.336	0.495	0.620	
*log*TI does not homogeneously cause *log*CO_2_	3.007 ***	5.359	0.000	*log*TI→*log*CO_2_
*log*CO_2_ does not homogeneously cause *log*TI	1.709	1.580	0.114	
*log*URB does not homogeneously cause *logCO_2_*	3.047 ***	5.476	0.000	*log*URB 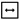 *log*CO_2_
*log*CO_2_ does not homogeneously cause *logURB*	2.102 ***	2.722	0.006	

Notes: *** *p* < 0.01.

## Data Availability

The data used in this study is publicly available at China Energy Statistical Yearbook (https://data.cnki.net/Yearbook, 20 May 2021), Chinese Provincial Statistical Yearbook (http://www.stats.gov.cn/tjsj/ndsj/, 20 May 2021) and World Development Indicator (https://databank.worldbank.org/, 20 May 2021).
